# Skeletal involvement in children with Langerhans cell histiocytosis: healing, complications, and functional outcome

**DOI:** 10.1051/sicotj/2020024

**Published:** 2020-07-16

**Authors:** Ahmed H. K. Abdelaal, Mohamed Sedky, Seham Gohar, Iman Zaki, Asmaa Salama, Omayma Hassanain, Ahmed M. El Ghoneimy

**Affiliations:** 1 Consultant of Musculoskeletal Tumor Surgery, Children Cancer Hospital-Egypt (57357) 11617 Cairo Egypt; 2 Lecturer, Department of Orthopedic Surgery, Faculty of Medicine, Sohag University 82524 Sohag Egypt; 3 Professor of Pediatrics, Pediatric Department National Research Centre, Consultant of Pediatric Oncology Children Cancer Hospital-Egypt (57357) 11617 Cairo Egypt; 4 Consultant of Pediatric Oncology, Children Cancer Hospital-Egypt (57357) 11617 Cairo Egypt; 5 Professor of Radiodiagnosis National Cancer Institute NCI, Cairo University Head of Medical Imaging Department, Children Cancer Hospital-Egypt (57357) 11617 Cairo Egypt; 6 Professor of Pathology National Cancer Institute NCI, Cairo University Consultant of Pathology, Children Cancer Hospital-Egypt (57357) 11617 Cairo Egypt; 7 Clinical Research Senior Supervisor, Children Cancer Hospital-Egypt (57357) 11617 Cairo Egypt; 8 Head of Musculoskeletal Tumor Surgery Unit, Children Cancer Hospital-Egypt (57357) 11617 Cairo Egypt; 9 Lecturer, Department of Orthopedic Surgery, Faculty of Medicine, Cairo University 12613 Giza Egypt

**Keywords:** Langerhans cell histiocytosis, Skeletal, Spontaneous remodeling

## Abstract

*Introduction*: Skeletal involvement in children with Langerhans cell histiocytosis (LCH) is a common feature of the disease. Several options for the treatment of these skeletal lesions have been reported. We describe our experience in the treatment of skeletal involvement of LCH in this retrospective case series study, entailing anatomic distribution, pattern of healing, skeletal deformities, and functional outcome of skeletal LCH. *Methods*: A retrospective analysis was conducted for patients diagnosed with LCH and having skeletal lesions in the period between 2007 and 2015. Out of a total of 229 cases, 191 (83.4%) had skeletal involvement. Bone healing was divided into partial and complete based on the size of lesion and cortical changes in plain radiograph. Skeletal deformities were serially measured. Time to pain control, resumption of weight bearing, and the final functional status of the patient were reviewed. *Results*: The mean age at presentation was 4.4 years (3 m–14.8 y) and the mean follow-up period was 53.3 months (0.2–120.7). After screening of skeletal and extra-skeletal lesions, 59 patients (31%) had M-S (Multisystem) LCH and 132 (69%) had S-S (Single system) LCH. Unifocal bone lesions were found in 81 (42.5%) patients, and multifocal bone lesions in 110 patients (57.5%). Single or multiple bone lesions were found in the craniofacial bones in 152 patients (79.5%), femur in 19 patients, (10%), ribs in 18 patients (9.4%), spine in 15 patients (8.1%), pelvis in 14 patients (7.3%), scapula in 8 patients (4.1%), humerus in 6 (3.1%), clavicle in 6 patients (3.1%), tibia in 3 patients (1.5%), radius in 3 patients (1.5%), and the ulna in 2 patients (1%) patients. No lesions were found in the fibula, hand, or foot. Out of all skeletal lesions, 179 (93.7%) patients were treated either medically or conservatively and 12 patients (6.2%) were treated surgically. The mean time to complete healing was 5.2 months (2–12). Skeletal complications included: pathologic fractures (9 vertebra plana, 5 long bone, 1 iliac bone), deformities (9 thoracolumbar kyphosis, 2 cervical spine subluxations, 2 coxa vara deformity of the proximal femur and one flattening of iliac bone). *Conclusion*: Non-operative treatment can lead to adequate bone healing in few months period. Partial or complete remodeling of bone deformities can be observed without surgical correction. However, surgical intervention might be indicated when cervical spine affection may lead to instability and subsequent neurological affection. Functional impairment is rarely caused by skeletal lesions in LCH.

## Introduction

Langerhans cell histiocytosis (LCH) is a rare disorder that is characterized by abnormal clonal proliferation of Langerhans-type dendritic cells [1]. It can affect several organs or systems in the body with skeletal system being the most frequently involved in around 80% of patients [2]. Histiocyte society categorized LCH into three types: single-system single-site (SS-s), single-system multiple-site (SS-m), and multisystem (MS). MS disease has been further subclassified into MS without risk organ involvement and MS with risk organ involvement [3, 4]. Although the prognosis is excellent for patients without risk organ involvement, twenty to thirty percent of patients with S-S bone disease developed permanent consequences, the majority of which are orthopedic complications directly related to the LCH bone lesion [2, 5].

Several options have been reported for the treatment for single bone lesions, including a simple biopsy and observation, oral treatment with indomethacin, systemic or local corticosteroid injection into bone lesions, curettage and bone grafting, and internal fixation to avoid fracture and deformity [6]. Most recent reports have shown adequate control of pain following simple biopsy and observation; however, few describe time to healing, possible deformities, and potential for remodeling which were the aims of the current retrospective analysis [7].

## Material and methods

A retrospective analysis was conducted for patients who were diagnosed with LCH in the authors’ institute. In the period between 2007 and 2015, medical records of 229 patients were reviewed. Diagnosis was confirmed histopathologically by immunophenotyping for CD1a or CD207 (langerin) with or without S100, according to the Histiocyte Society criteria [3]. A biopsy was performed in all patients with the exception of 10 patients for which no histopathologic diagnosis could be found in their medical records and 2 patients for which a biopsy was not technically feasible. In the remaining 217 patients, 59 patients were referred to hospital with already performed biopsy and 158 patients did bone or soft tissue biopsy in the authors’ institute. It is reported in the literature that when the risk of biopsy outweighs the need for a definitive diagnosis, biopsy could be skipped and treatment plan will be applied with close monitoring [3, 4].

Radiological assessment included a plain radiographic skeletal survey, plain anteroposterior chest radiograph, and abdominal ultrasound. Further imaging studies such as computed tomography or magnetic resonance imaging were done on a case-by-case basis.

From a total of 229 patients, 38 patients (16.6%) had no skeletal involvement. These patients were excluded from the current study, leaving a final number of 191 patients (83.4%) who were subjected to analyses. The medical records of the latter group of patients were reviewed for age, gender, follow-up duration, skeletal distribution and number of lesions, radiographic time to healing and remodeling, complications such as fracture or deformity, and functional outcome. The first visit for patients in the orthopedic clinic was generally held between week 3 and 4 from diagnosis. Further follow-up was at 3 monthly intervals until complete healing of bony lesions and thereafter every 6 months for the following 2 years.

To define the quality of bone healing, we devised a radiographic staging system for skeletal lesions based on two criteria: cortical healing or remodeling and the decrease in size of lytic area (Table 1). Healing was classified accordingly into partial (PH) and complete (CH). Any appreciable deformity in plain radiographs was measured and followed for remodeling or progression throughout serial radiographic evaluations done for each patient. Functional evaluation was based on the following criteria: time to pain improvement, period of immobilization, time to return to activity since diagnosis, and the presence of any clinically recorded disability and its progression until latest clinical evaluation of the patient.

**Table 1:** Stages of bone healing.

Stages of healing	Size of lytic area	Cortical remodeling
Partial healing (PH)	Decrease in size of intraosseous lysis	Quiescence of periosteal reaction/consolidation of cortex
Complete healing (CH)	Complete disappearance of lytic area in bone	Return to normal shape of bone

## Results

The mean age at presentation was 4.4 years (3 m–14.8 y) and the mean follow-up period was 53.3 months (0.2 m–120.7 m). There were 110 male and 81 female patients. Among a total 191 patients with skeletal involvement, 59 (31%) were part of M-S LCH, and 132 patients (69%) had S-S disease with the skeletal system as the only involved organ. Among S-S LCH, 67 patients (51%) had a unifocal bone lesion and 65 patients (49%) had multifocal bone lesions. Among M-S LCH, 14 patients (24%) had single bone lesion, and 45 patients (76%) had multiple bone lesions. Table 2 shows the number and frequency of skeletal lesions according to their anatomic distribution, survival rates of different case categories as well as age at presentation and follow-up duration.

**Table 2:** Descriptive statistics of study cases, classification of patients according to histiocytosis Euro Network, survival, age at presentation, and follow-up duration.

Category	Number of cases (percentage)	Number of died cases	Survival	Age at diagnosis in years	Follow-up period in months
				Mean ± SD (range)	Mean ± SD (range)
LCH not affecting the skeletal system	38	Excluded from the study			
Multisystem LCH					
Multifocal bone lesions	45	9	80%	2.8 ± 2.23 (0.3–10.25)	43.4 ± 33.5 (8–118.8)
Unifocal bone lesion	14	2	85%	30.4 ± 3.1 (0.4–10.1)	50.3 ± 40.4 (30–119)
Skeletal-only LCH					
Multifocal bone lesions	65	1	98.5%	4.27 ± 3 (0.48–12.36)	61.8 ± 31 (20–120)
Unifocal bone lesion	67	2	97%	6.08 ± 3.6 (0.44–14.84)	53.5 ± 32.3 (1.5–120.8)
Total	191	14	92.7%	4.4 ± 3.2 (0.3–14.84)	53.3 ± 33.3 (1.5–120.8)

### Axial skeleton

#### Craniofacial region

Craniofacial region is the most commonly involved part of the skeleton in 152 patients (79.5%). Skull lesions were solitary in 78% of patients. The parietal bone was most common site in 33% of patients, followed by the frontal bone in 14%. The orbit was involved in 10% of the craniofacial bones and the mandible in 3%. Out of the total craniofacial lesions, 27 (17.7%) had an open biopsy and 9 patients (5.9%) were locally treated by excisional biopsy (5 skull, 2 orbital, and 2 mandibular lesions). No patient ocular or neurologic complications were recorded secondary to a nearby bone lesion.

#### Spine

**Figure 1:**
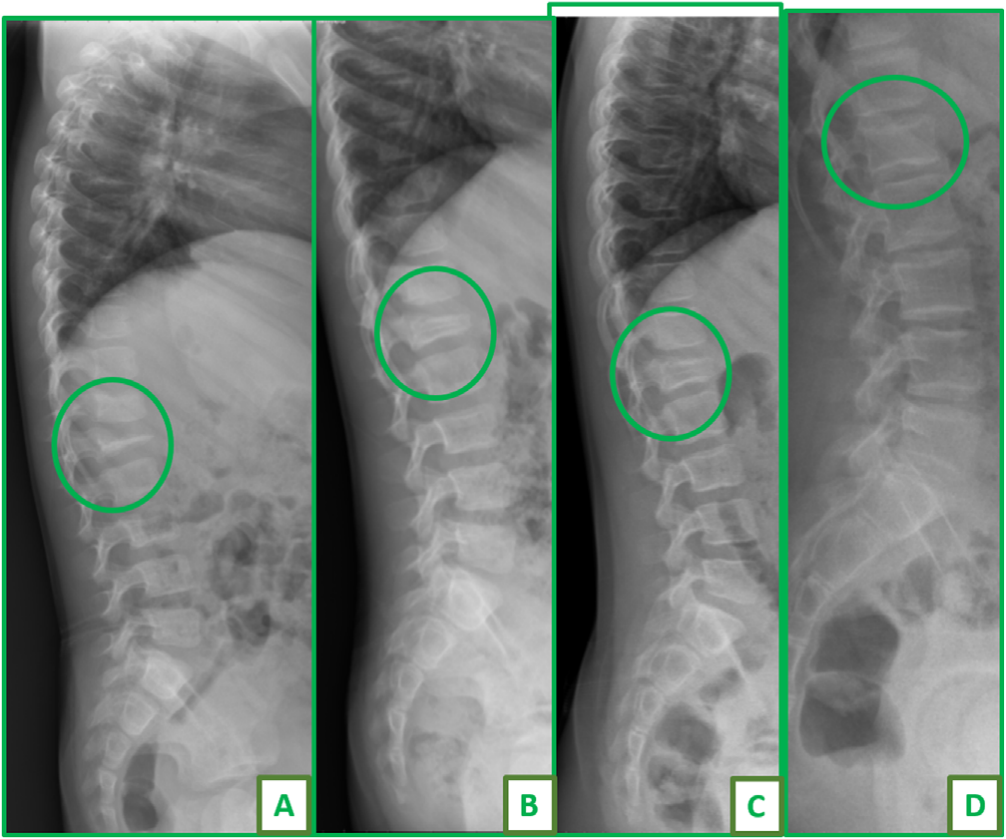
Plain radiograph of a skeletal lesion in the lumbar spine L1 of a 3-year-old boy managed conservatively. (A) Marked flattening of L1, vertebra plana. (B) Gradual remodeling and restoration of the height after 1-year follow-up. (C) Remodeling after 2 years. (D) Complete restoration of height after 5-year follow-up, child age 8 years.

**Figure 2:**
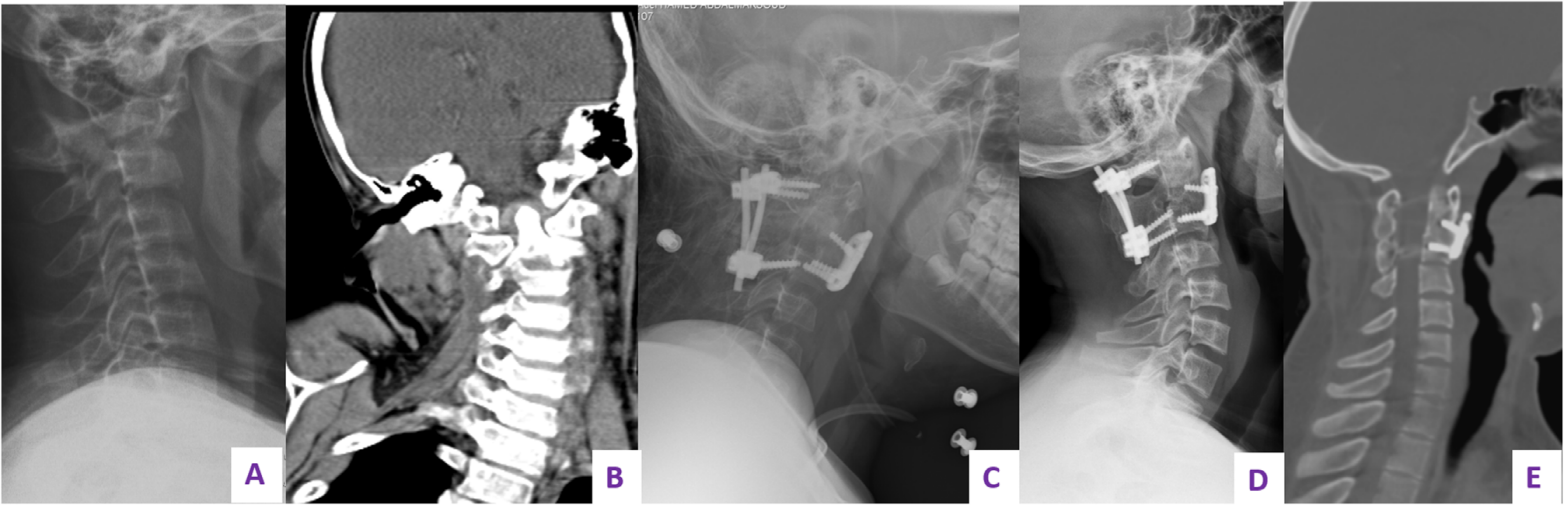
Plain radiograph of cervical spine of a 12-year-old boy diagnosed with LCH showing destruction and atlanto-axial instability, surgery was mandated to avoid neurological affection. (A) Preoperative plain radiograph showing C1–C2 instability. (B) CT scan showing the instability and lateral tilt of C1 over C2. (C) Postoperative radiograph showing C1–C2 fixation. (D) Follow-up radiograph after 1 year. (E) Follow-up CT scan after 5 years showing complete fusion of C1–C2.

Spine was involved in 15 patients (8.1%). In order of frequency, the dorsal vertebrae were the most commonly involved in 8 patients, followed by the cervical spine in 5 patients and the lumbar spine in 4 patients. Two patients had combined lumbar and dorsal spine affection and one patient had combined cervical and dorsal spine involvement. Apart from two patients who presented with anterior wedging of the involved vertebrae, 13 patients had complete flattening of the affected vertebrae with a resulting thoracic kyphosis and a mean Cobb’s angle of 44° (34°–74°) in 8 patients and subluxation at the level of C1–C2, C4–5, and C7–T1 in three patients. All patients with thoracic and lumbar involvement were treated conservatively using thoracolumbar support and follow-up. Partial restoration of the vertebral height to greater than 50% was noticed in 10 patients after a mean follow-up duration of 49 months (36–84). Figure 1 shows spontaneous complete restoration of vertebral height and complete remodeling in first lumbar vertebra in a 3-year-old girl. Two patients died of disease 26 and 28 months since the date of first presentation and had persistent flattening of the involved vertebrae in their latest radiographic evaluation. Among patients with subluxation of cervical spine, none had any neurologic deficit due to the existing instability neither at presentation nor at their latest follow-up visit. Two patients were surgically treated by anterior and posterior fixation at the level C1–C2 and C7–T1 (Figure 2). One patient with flattening and subluxation at the level of C4–C5 was treated conservatively with spontaneous reduction of the subluxation and partial restoration of the vertebral height at 48 months of follow-up.

#### Pelvis

Pelvis was involved in 15 patients (7.8%). The most commonly affected part of the hemipelvis was the ilium in 6 patients, followed by the acetabulum in 4 patients and the inferior pubic ramus in 1 patient. Four patients had diffuse pelvic infiltration as part of their M-S disease. All patients were treated conservatively without any additional surgery apart from the open biopsy. Complete healing developed at a mean of 9.3 months (3–20). None of the patients with acetabular lesions develop any acetabular deformities or joint damage and all had a normal range of motion of their hips at their latest follow-up.

### Appendicular skeleton

#### Femur

**Figure 3:**
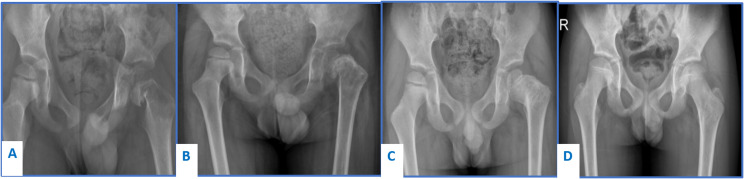
Plain radiograph of the left hip of a 5-year-old boy diagnosed with LCH in the femoral neck managed conservatively. (A) Pathologic fracture of the left femoral neck on top of lytic lesion. (B) Healing of the fracture after 3 months. (C) Remodeling of the femoral neck at 1 year of follow-up. (D) Complete remodeling after 7-year follow-up.

Femur was involved in 19 patients (11.1%). This included 12 patients with S-S LCH and 7 patients with M-S LCH. All S-S unifocal lesions were diaphyseal located, while 4 out of 7 multifocal lesions were in the proximal femur. Out of a total of 19 patients, 3 (15.7%) presented with pathologic fractures, 2 in the proximal femur with coxa varus deformity of the femoral neck, and one pathologic fracture of the midshaft. Eighteen patients were treated conservatively by immobilization in cast until the consolidation of the femoral cortex at a mean of 2.7 months (2–4). One patient was treated by open curettage of the lesion and filling of the bone cavity with bone graft substitute. All lesions showed radiographic evidence of complete healing at a mean of 7.5 months (3–12). Plain radiographic evidence of complete remodeling of the femoral neck and correction of the varus deformity was observed one year following presentation, in a 4-year-old child who presented with fracture and coxa vara angulation of the femoral neck (Figure 3). Another 7-year-old patient with coxa vara of the femoral neck was lost to follow-up at 7 months from presentation and his last plain radiographs showed healing of the lesion but a persistent minimal coxa vara deformity.

#### Tibia

Tibia involved in 3 patients. All were diaphyseal lesions and no patient presented with or developed any pathologic fracture or deformity during their follow-up. All lesions healed conservatively following an open biopsy.

#### Shoulder girdle

The humerus was involved in 6 patients. Three lesions were located in the proximal humeral metaphysis, two in the distal humerus, and one lesion was diaphyseal. All treated conservatively and healed without any fractures or deformities.

The scapula was involved in 8 patients (4 in the glenoid area, 3 involving the whole scapula and 1 lesion was localized to infraspinatus fossa). Five lesions were large in size ballooning the whole scapula but all were treated conservatively with complete healing and full range of motion of the shoulder joint at their latest follow-up.

The clavicle was involved in 6 patients and was treated conservatively without any resulting fractures or deformities.

#### Forearm

**Figure 4:**
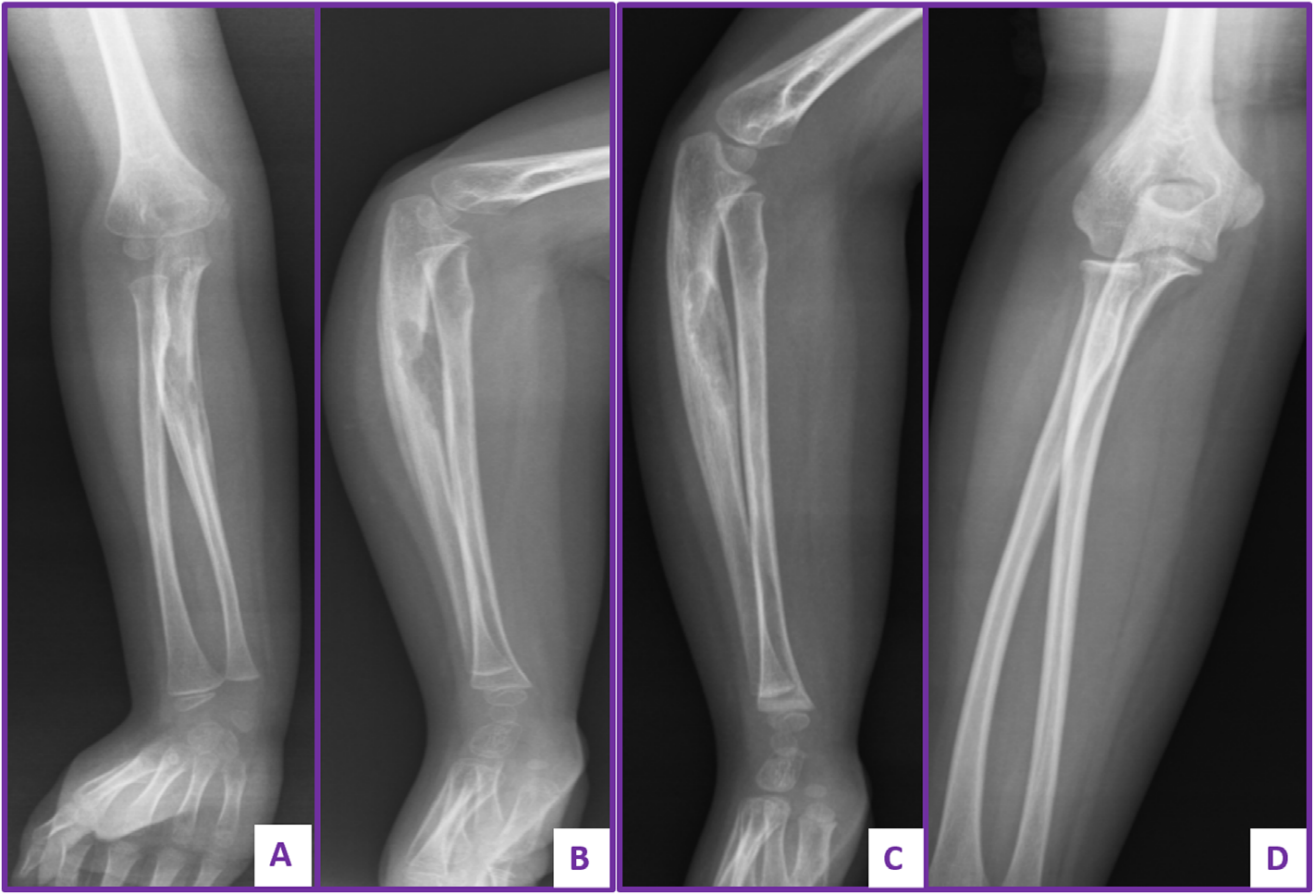
Plain radiograph of forearm of a 5-year-old girl diagnosed with LCH showing large osteolytic lesion of the ulna managed conservatively. (A and B) Anteroposterior and lateral views showing the large sized osteolytic lesion of the ulna. (C) Follow-up after two years showing partial remodeling. (D) Follow-up radiograph showing complete remodeling.

The radius was involved in three and the ulna in two patients. Three lesions resulted in marked expansion of the proximal part of involved radius and ulna reaching the articular surface; however, all were treated conservatively with complete healing and full range of motion of the elbow joint (Figure 4). No lesions were recorded in hand or foot bones.

## Discussion

The reported survival rate for patients diagnosed with S-S and M-S risk organ negative LCH are over 90% [1, 2, 7]. In our case series, survival rate was about 100% in M-S risk organ negative LCH patients, though in all M-S patients it was about 84%, as all eleven died cases from M-S patients were risk organ positive cases. Survival rate was much better among skeletal system only LCH patients either single or multifocal cases. Various local treatment modalities are described in literature including, chemotherapy, radiotherapy, simple biopsy and observation, oral intake of indomethacin, local intralesional steroid injection, excisional biopsy or curettage and bone grafting [1, 8]. Recent reports showed that good results can be obtained following biopsy and follow-up of bone lesions [9]. Rivera et al. analyzed resolution of symptoms in 39 S-S unifocal skeletal lesions. The median time from biopsy to resolution of symptoms was 5.43 weeks and there was no significant difference among patients who received incisional biopsy, trocar biopsy or biopsy with grafting. They concluded that unifocal osseous lesions likely do not require aggressive surgical management [6]. The same conclusion was reached by Sasaki et al.; however, they stated that the optimal follow-up period is not yet established, and this should be a focus of studies in the future [7]. In the current study, our results are consistent with previous reports showing adequate healing of most of the lesions following a simple biopsy, but our results are more location specific, we suggested the partial healing concept as a tool to allow gradual return to full functional activity, as partial healing is mostly accompanied by improvement of pain which significantly affects the child physical activity. Time needed for these lesions to heal either partially or completely was analyzed for each anatomic location. Most radiographs showed evidence of partial healing at 2.3 months (1.5–5) by which time the patient was allowed progressive return to activity and complete healing at 7.2 months (2–25). Restriction of activity until complete healing was not done in any patient and no patient showed evidence of fracture or deformity secondary to earlier mobilization. It is noteworthy that spine involvement takes obviously longer time till restoration of the vertebral height and remodeling, restoration of more than 50% of vertebral height was considered as partial healing, though pain control was achieved at much earlier time. In two cervical cases with instability at C1–C2 and C7–T1 levels, we were afraid of progressive deformity or occurrence of neurological deficit, thus we proceeded to fixation and fusion, though in another case with instability of C4–C5 level was managed conservatively using a rigid cervical collar and close follow-up with spontaneous improvement and restoration of vertebral height at 48 months, This was consistent with the findings of Dormans et al. on their series of patients with spinal LCH. They stated that no patient had neurologic impairment and concluded that most patients can be treated conservatively without the need for surgery [10].

Pelvic involvement shows an excellent outcome even with diffuse infiltration or acetabular involvement, Remodeling of pelvic bones occurs at a longer time tan craniofacial or long bones, but eventually with minimal, if any morbidity. No persistent deformity, pathological fracture, or other complication was reported. Long bone lesions heal at a faster rate than pelvic ones, femoral lesions which constitute the majority of long bone affection healed partially with return to physical activity in a mean of 2.7 months, in only one of earlier cases we did curettage and used bone substitute to fill the cavity. Even in peritrochantric area and femoral neck, conservative treatment yields satisfactory results; Figure 3 shows the gradual remodeling of coxa vara deformity over a period of 12 months, complete restoration of the neck shaft angle was noted about 48 month duration. Tibial, humeral, radial, and ulnar lesions had healed at nearly comparable durations and faster than femoral lesions do; due to a relatively smallr number of cases, we could not run a statistical test to show the significant difference between weight bearing and non-weight bearing bones, or between flat bones and long bones. We recommended protected weight bearing in lower limb or splinting in upper limb is applied till partial bone healing is radiologically evident. Comparing our results to local corticosteroid injection done by Mavrogenis et al., they had mean time to complete bone healing of 1 year (8–14 months), which demonstrates that local corticosteroid injection did not hasten the time to bone healing as compared to simple biopsy and observation [8]. Braier et al. used Indomethacin for treatment of symptomatic bone lesions in 38 LCH patients. The median duration of treatment was 3 months and 81% of patients had non-active disease at 8 weeks of treatment. They stated that bone healing was slow and was not in parallel with the disease response and that their cutoff point of 8 weeks may be insufficient to evaluate the response of bone lesions and therefore the optimal duration of therapy was difficult to establish and was not uniform in all their patients [11]. As previously mentioned, in the current analyses, the mean time to partial bone healing was 2.3 months which is consistent with their 8-week-cutoff point for assessment of treatment response and indicates the insignificant difference between simple biopsy and Indomethacin treatment in terms of disease control (Table 3).

**Table 3:** Anatomic distribution of skeletal lesions, time to partial and complete healing.

Bone involved	Frequency in % (number of patients)	Time to PH in months	Time to CH in months
		Mean (range)	Mean (range)
Craniofacial	79.5% (152)	2.3 (1.5–5)	
Spine	7.8% (15)	49 (36–84) restoration of >50% of vertebral height	
Weight bearing bones			
Femur	10% (19)	2.7 (2–4)	7.5 (3–12)
Tibia	1.5% (3)	3 (2.5–4)	7 (6.5–8)
Pelvis	7.8% (15)	3.4 (1–12)	9.3 (3–20)
Non weight bearing bones			
Scapula	4% (8)	3.8 (2.5–6.5)	5.5 (4.3–6.7)
Clavicle	3% (6)	2.8 (2.5–4)	4.8 (3.5–5.5)
Humerus	3% (6)	2.7 (2–4.5)	5.2 (3.5–7)
Radius	1.5% (3)	2.7 (2–3.5)	5.7 (4–6.5)
Ulna	1% (2)	3 (2.5–3.5)	5 (4.5–5.5)

We also analyzed skeletal deformities and their course throughout follow-up of patients and we found that almost half of the deformities are manifested in the form of bone expansions without any deleterious biomechanical effect in spite of the stationary course of these expansions after bone healing. The other half of patients who developed skeletal deformities had angular malalignment secondary to pathologic fractures, or vertebra plana cases which were gradually remodeled; iliac bone flattening had occurred in one case, and spontaneously improved. The majority of these patients were conservatively treated and over 90% showed partial remodeling of the deformity without any functional impairment at their latest follow-up visit.

## Conclusion

Spontaneous improvement of the skeletal involvement of LCH lesions is expected in majority of cases even after a simple core or open biopsy which can lead to healing and partial remodeling of bone in a time frame comparable to that previously reported with other lines of treatment. This conservative management avoids unnecessary surgeries or medications with their possible associated risks or side effects. In addition, the demonstrated relatively high ability of the bone to remodel in these young LCH patients compared to adult population, encourages the usefulness of watchful observation of skeletal deformities and reservation of surgical interference for the few recalcitrant cases which do not show evidence of healing and or are associated with functional impairment.

## Conflicts of interest

All authors confirm that they did not receive any internal or external fund for the presented work. All authors have no conflicts of interest to declare.
